# Knowledge and acceptability of the human papillomavirus vaccine among health professionals in Acre state, western Amazon

**DOI:** 10.6061/clinics/2019/e1166

**Published:** 2019-09-26

**Authors:** Julio Eduardo Gomes Pereira, Jéssica Menezes Gomes, Annielson de Souza Costa, Francisco Winter dos Santos Figueiredo, Fernando Adami, Edige Felipe de Sousa Santos, Isabel Cristina Esposito Sorpreso, Luiz Carlos de Abreu

**Affiliations:** I Centro de Ciencias da Saude e do Desporto, Universidade Federal do Acre, Rio Branco, AC, BR; II Disciplina de Ginecologia, Faculdade de Medicina FMUSP, Universidade de Sao Paulo, Sao Paulo, SP, BR; III Laboratorio de Epidemiologia e Analise de Dados, Faculdade de Medicina do ABC, Santo Andre, SP, BR; IV Departamento de Epidemiologia, Faculdade de Saude Publica, Universidade de Sao Paulo, Sao Paulo, SP, BR; V Programa de Mestrado em Politicas Publicas e Desenvolvimento Local da Escola Superior de Ciencias da Santa Casa de Misericordia, Vitoria, ES, BR.; VI Laboratorio de Delineamento de Estudos e Escrita Cientifica, Centro Universitario Saude ABC, Santo Andre, SP, BR.

**Keywords:** HPV, Papillomavirus Vaccines, Knowledge, Health Professional

## Abstract

**OBJECTIVE:**

To evaluate the knowledge and acceptability of the human papillomavirus (HPV) vaccine among health professionals from western Amazonia.

**METHODS:**

A cross-sectional study was conducted in the Sistema Assistencial à Saúde da Mulher e da Criança (Health Care System for Women and Children; SASMC) in Acre, Brazil, in 2017. The participants comprised 196 health professionals. The data collection instrument contained 31 questions about HPV, its clinical repercussions for women, and the HPV vaccine. Quantitative variables were presented as medians and 95% confidence intervals (CIs), and *p*<0.05 was considered statistically significant. For the analyses, chi-square tests and Mann-Whitney tests were used. The collected data were analyzed using Stata®11.0.

**RESULTS:**

Of the 196 health professionals, 39.8% (n=76) were physicians and 61.2% (n=120) were other health professionals. The interviewees were mostly female (n=143, 73%, 95% CI 66.1 to 78.9%) who worked in the medical field (n=81, 41.3%, 95% CI 34.4 to 48.6%), and the median age was 38 years (95% CI 36.0 to 39.7). Physicians had increased knowledge regarding only the statement "cervical cancer is one of the main causes of cancer in women", with a proportion ratio of 0.88 (0.80; 0.97) and *p*<0.001. Regarding clinical knowledge of the HPV vaccine, a low proportion of correct answers was obtained for all the questions, and no significant differences were found between the groups.

**CONCLUSION:**

Acceptability and knowledge of HPV and its vaccine were similar among health professionals, with knowledge gaps in questions about the relation between smoking and cervical cancer and specific clinical knowledge.

## INTRODUCTION

Human papillomavirus (HPV) infections of the genital tract 1 are related to cervical cancer. Thus, HPV is the third most prevalent cause of tumors and the fourth leading cause of death due to cancer in Brazil 2. The persistence of oncogenic HPV subtypes, especially 16 and 18, is related to more than 80% of cervical cancer cases 3 as well as other cancers. Conversely, subtypes 6 and 11 are nononcogenic and are associated with genital warts 4.

The primary prevention strategies for HPV infection are vaccination and condom use. The quadrivalent vaccine (Gardasil TM) is composed of noninfectious antigenic particles capable of inducing a protective immune response 5,6, and the target populations for the quadrivalent HPV vaccine are girls 9 to 14 years of age and boys 11 to 14 years of age 7. Both are available from the Brazilian health care system at no cost.

The World Health Organization (WHO) considers vaccination coverage adequate when inoculations of the second dose reach 80% in the target populations 1. In Brazil and the Federative Unit of Acre, 100% adherence was achieved for the first vaccine dose, and 64.48% and 49.84% adherence rates were achieved for the second doses in girls and boys in 2017, respectively 8.

Acceptability of the vaccine contributes to adherence in vaccination campaigns and is influenced by social, economic, and cultural factors as well as the population's confidence in its safety and efficacy 9. Health professionals affect the decision-making of patients and guardians regarding vaccination 10-12 by minimizing vaccination barriers and increasing the acceptability of the prevention method through cultural guidance and demystification 13.

Territorial studies can contribute to improving public health by helping identify gaps in knowledge among health professionals and thus promoting territorial health education. The objective of this study was to evaluate the knowledge and acceptability of the HPV vaccine among health professionals in the western Amazon.

## METHODS

### Study Design

This cross-sectional study was conducted in the Sistema Assistencial à Saúde da Mulher e da Criança (Health Care System for Women and Children; SASMC) in the state of Acre, Brazil, from January to March 2017.

Acre state is a Brazilian Federative Unit localized in northern Brazil and has a territorial extension of 164.221,36 km^2^ (2% of Brazilian territory, approximately); Acre had a Human Development Index of 0.66 (Brazil was 0.72) and a Gini index of 0.63 (Brazil was 0.60) in 2010. One hundred percent adherence was achieved for the first vaccine dose and 78.48% and 42.1% adherence rates were achieved for the second doses in girls and boys in 2017, respectively 8.

### Population

Physicians and health professionals of both sexes who were over 18 years of age and participated in the study were enrolled through convenience sampling. All participants signed the Free and Informed Consent Form, and the study was approved by the Research Ethics Committee of the Faculdade de Medicina da Universidade de São Paulo (School of Medicine of the University of São Paulo) - USP (205/14) and the União Educacional do Norte (North Education Union) - UNINORTE (2.158.359).

A pilot study with 20 individuals was performed to identify the parameters required to calculate the sample size. The statistical parameters used were as follows: the means (standard deviations (sds)) of the overall score between physicians (85.4; sd=5.4) and other health professionals (80; sd=17.6), a test power of 80%, and a standard error of 5% for a bilateral test. A sample loss of 20% (n=32) was considered, resulting in an estimated size of 192 individuals.

### Data Collection Procedure

The collection instrument for the evaluation of knowledge and acceptability of the HPV vaccine among health professionals contained a sociodemographic evaluation (sex, marital status, number of children, profession, family income, schooling, and type of higher education). The instrument was composed of 31 questions, including 24 questions covering HPV knowledge topics, HPV vaccine knowledge, HPV vaccination barriers, and HPV vaccine acceptability; 3 (three) questions on personal history related to cervical cancer and genital warts in female subjects; and 4 (four) questions about the HPV vaccine in special situations (pregnancy and immunocompromised patients) 14.

The response options for the instrument questions were yes, no, and not sure. The questions were grouped by theme to score the answers, with one point assigned to each question answered correctly. The scores were inverted for questions 11, 12, 19, and 31.

The overall score weighting was calculated from the 24 questions covering knowledge topics about HPV, the vaccine, barriers to vaccination, and acceptability of the HPV vaccine. Questions 1, 15, 22, 23, 25, 26, and 27 were considered personal responses and were not counted in the overall score.

A good level of knowledge and acceptability was considered when the mean overall score was equivalent to 80% correct answers; the score was calculated as the sum of correct answers divided by the total number of questions, as shown in the formula below:


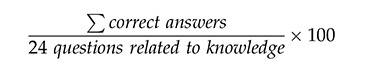


### Data Analysis

The data collected were tabulated using a Microsoft Excel spreadsheet. Qualitative variables were presented as absolute and relative frequencies with their respective 95% confidence intervals (CIs). Quantitative variables were presented as medians and 95% CIs because they did not have normal distributions (evaluated by the Shapiro-Wilk test, *p*<0.05). To analyze the differences in characteristics between physicians and other health professionals, the chi-square test was used for qualitative variables, and the Mann-Whitney test was used for quantitative variables.

Poisson regression with robust variance adjusted for sex, stable union, age, and income was used to estimate the proportion of correct answers to questions related to HPV knowledge between the groups. Interquartile regression adjusted for sex, stable union, age, and income was used to analyze the median difference in the overall HPV knowledge scores between groups. The confidence level used was 5%, and the software used for the analysis was Stata® (StataCorp. 2009. Stata Statistical Software: Release 11. College Station, TX: StataCorp LP).

## RESULTS

A total of 196 health professionals, consisting of 39.8% (n=76) physicians and 61.2% (n=120) other health professionals, were divided into two groups and interviewed. The professionals were predominantly female (n=143, 73%, 95% CI 66.1 to 78.9%) with a stable union (n=109, 58.6%, 95% CI 51.1 to 65.7%) and children (n=130, 67.4%, 95% CI 60.2 to 73.8%), and nearly half were physicians (n=81, 41.3%, 95% CI 34.4 to 48.6%). Overall, the median age of the sample was 38 years (95% CI 36.0 to 39.7), and the respondents had a median of 2 children.

The ages and number of children did not differ between the groups (assessed by the interval estimate and 95% CI of the proportions). The proportion of individuals with an income above 10 wages was significantly higher in the group of physicians than in the group of other professional (*p*<0.001) (Table 1).

A knowledge gap with a proportion of 71.9% (95% CI 65.0; 78.0) was identified among all health professionals in response to the question "Smoking may increase the risk of cervical cancer", with no difference observed between the groups (Table 2).

According to the parameters used in the study, a low proportion of correct answers regarding the HPV vaccine was found for the following questions: "Can the HPV vaccine be given to those who have had sexual intercourse?" (74.0%, 95% CI 67.1; 79.9); "Can the HPV vaccine be harmful to health?" (77.6%, 95% CI 70.9; 83.1); "Can the HPV vaccine cause infection?" (75.0%, 95% CI 68.2; 80.8); "Is the HPV vaccine part of the girls' immunization records?" (73%, 95% CI 66.1; 78.9); "Is it necessary to take 3 doses for complete vaccination?" (61.2%, 95% CI 54.0; 68.0); and "Does the HPV vaccine decrease the odds of contracting genital warts?" (71.9%, 95% CI 65.0; 78.0) (Table 2).

In the analysis between physicians and other health professionals, increased knowledge of the physicians was observed regarding only the statement "Cervical cancer is one of the main causes of cancer in women", with a proportion ratio of 0.88 (0.80; 0.97) and *p*<0.001 (Table 2).

The perceptions regarding barriers to vaccination and acceptability were not different between the physicians and other health professionals, as observed by the CIs of the interval estimates of the proportion ratios and their respective p values. The personal backgrounds of the female professionals presented a relatively low proportion of nonmedical professionals; 29% (PR: 0.71; 95% CI 0.29 to 1.74) had little knowledge about abnormal Pap smear results and 83% (PR: 0.17; 95% CI 0.03 to 1.12) demonstrated little knowledge about a genital warts report (Table 3).

Regarding clinical knowledge of the HPV vaccine, a low proportion of correct answers was found for all questions, and no significant differences were found between the groups (Table 4).

**Figure 1:**
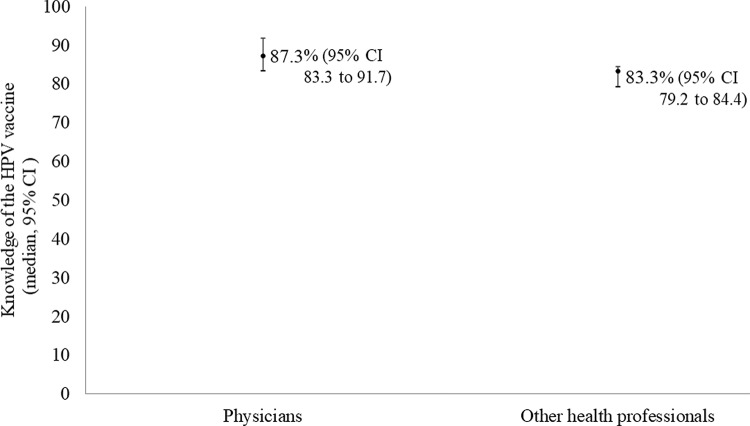
General knowledge of the HPV vaccine among physicians and other health professionals interviewed in the Health Care System for Women and Children (SASMC) in the state of Acre, Brazil, in 2017. Interquartile regression*: β=-4.2 (95% CI -13.7 para 5.3); *p*=0.10 *Adjusted by sex, stable union, age, and income.

Among physicians, the proportion of correct answers regarding general knowledge of HPV, its clinical repercussions, and the HPV vaccine was 87.3% (range from 83.3 to 91.7%), whereas among the other health professionals, this knowledge was 83.3% (range from 79.2% to 84.4%). The difference was not significant when adjusted for sex, stable union, age, and income (Figure 1).

## DISCUSSION

The analysis of health professionals in the western Amazon who were interviewed about their knowledge of the HPV virus, its repercussions for the health of women, and its vaccine showed a knowledge gap concerning smoking as a cofactor for the development of cervical cancer and a low proportion of correct answers regarding HPV vaccine safety, efficacy, and the vaccination schedule.

The interviewees were female, worked in the medical field, and had stable unions and children. Cervical cancer affects women who are in an economically active period of their life. Since the interviews were conducted mainly with those in the medical field, knowledge of the topic is justified.

The concept of health has been transformed on the basis of historical experience, resulting in the appearance of new formulations in public health and consequently new proposals for changes in healthcare models. Vaccination for HPV is an example of this change since its inclusion in the vaccination program was necessary to solve the high rates of uterine cervix cancer 15.

No barriers to vaccination were identified among the respondents. However, the cost of the vaccine and anticipation of the onset of sexual activity 16,17 are the most cited items in the literature and are related to sexual myths or prejudices regarding the vaccine. This finding shows the need for clarification on the subject among the interviewees who work in adolescent health in the studied region.

The high number of correct answers regarding the need for continued Pap smear procedures (secondary prevention for cervical cancer) identified in this study suggested knowledge of the importance of screening and early detection of cervical cancer since the primary prevention offered by the quadrivalent HPV vaccine showed a reduction in the numbers of low- and high-grade premalignant lesions identified by the Pap smear test 18.

We emphasize the need for adequate knowledge of HPV, its repercussions, and its relationship with cervical cancer among health professionals. Smoking as a nonviral factor associated with cervical cancer was considered a gap in knowledge among the interviewees. Smoking is a risk factor for the development of invasive cancer and cancer *in situ* 19. Moreover, the risk of squamous cell carcinoma increases with the number of cigarettes smoked per day and the onset of smoking at an early age 19,20. Incentives for smoking cessation in health promotion programs and healthy lifestyle guidelines for the young population should be included in health education by professionals.

HPV infection of the genital tract is considered the most frequent viral infection among sexually active individuals 21. The perception of HPV as a sexually transmitted infection among health professionals has been satisfactory. Studies 22,23 conducted with similar groups have shown a lack of knowledge about the sexual transmission of HPV according to the professional's area. Knowledge about the viral etiology of HPV, its causal relationship with genital warts, changes in Pap smear tests, and its association with cervical cancer was similar among the professionals interviewed.

Health professionals have doubts about the vaccination schedule advocated by the National Immunization Program. Gaps in knowledge or doubts about the vaccination scheme among health professionals have been shown to be similar in developed and developing countries 12,23,24. Recommendations for health professionals reinforce the validity of the immunization program and increase vaccination coverage 22.

The National Immunization Program indicates vaccination for HPV in men and women with HIV from 9 to 26 years of age; this is a different population and a different age range from those targeted in the campaign for HPV vaccination among adolescents 7. These differences may contribute to confusion among health professionals.

The existence of several HPV vaccines (bivalent, quadrivalent, and nonavalent) 25 as well as the adoption of different vaccine schedules in countries that offer the HPV vaccine free of charge may be confounding factors for understanding the vaccination schedule among health professionals.

The interviewees were not sure about the target or specific population that should be vaccinated. Criteria for vaccination in women living with HIV as well as contraindications in pregnant women should be emphasized in continuing education as topics for health professionals.

The knowledge gap regarding vaccine indications in HIV-positive individuals arises from misinformation about the safety of the HPV vaccine in immunosuppressed patients. The vaccine is safe in patients living with HIV and may be given after a medical prescription because it contains no live or attenuated virus but instead contains proteins without viral particles 7,25-27.

A low level of correct answers about the nonuse of the HPV vaccine in pregnant women was observed in the present study. The HPV vaccine is not recommended for pregnant women and is not part of the vaccine schedule for this population. However, epidemiological surveillance studies have indicated the nonteratogenicity of the vaccine 28,29.

According to the Ministry of Health's vaccine data, vaccine coverage was 100% for the first dose in the state of Acre (in western Amazonia); importantly, vaccine coverage above 80% is considered adequate 7. The interviewees showed acceptance and willingness to recommend the HPV vaccine, verifying the adequate vaccination coverage in the state of Acre, which is positively related to acceptance of the HPV vaccine among health professionals 11-13. Acceptance in this group of professionals was similar in countries where the vaccine was adopted in public health programs 30,31.

In a recent WHO review on the HPV vaccine, recommendations were given to the scientific community regarding the importance of encouraging the second dose and surveillance in the target population because better outcomes are obtained in reducing cervical cancer percussive lesions and producing immune responses when the second dose is completed 1. In Brazil, vaccination coverage for the second dose of the HPV vaccine was 44.65% in 2015 and was 42.02% in the state of Acre, reinforcing the importance of health education on this topic for professionals in this state 8.

Despite good acceptance, some professionals said they were not vaccinated for HPV. The National Immunization Program of the Ministry of Health does not include health professionals as a target population, and the age group does not correspond to the target population for vaccination 7. The contribution of health professionals to adequate vaccination coverage in our state can be broadened through the implementation of continuing education strategies for professionals and can thus fill the knowledge gaps found in this study.

Knowledge regarding HPV among the health professionals in our study was similar among both medical graduates and graduates from other health science courses. In contrast, with our results, Nilse et al. 32 reported that nurses working in public health had more knowledge about the topic than physicians. Furthermore, a study conducted in the United Kingdom and Ireland showed that health professionals in general had good knowledge of HPV 33.

The acceptability of the HPV vaccine among health care professionals in Acre was satisfactory based on our results, with knowledge gaps among respondents regarding vaccine-specific questions. Nurses, speech therapists, and other health care professionals had more questions about specific HPV vaccine factors than did oncologists 33.

Studies with a cross-sectional design are subject to reverse causality (i.e., the impossibility of distinguishing the temporality between the levels of knowledge and the acceptability of the HPV vaccine among health professionals). External validity (the generalization of the results to other regions of Brazil) is also a limitation because the population is a convenience sample of health professionals in Acre.

Thus, the findings of this study showed knowledge gaps regarding the nonviral cofactors related to cervical cancer (smoking), the vaccination schedule, and the eligibility of immunocompromised populations to receive the vaccine. The topics of smoking and cervical cancer, the vaccination schedule for the quadrivalent HPV vaccine, and the recommendations for vaccination of individuals living with HIV should be included in continuing education for health professionals.

## CONCLUSIONS

Acceptability and knowledge of the HPV vaccine were similar among medical and nonmedical health professionals. Knowledge gaps were found regarding the vaccination schedule, vaccine use among immunocompromised individuals and pregnant women, and the involvement of smoking in the etiology of cervical cancer.

## AUTHOR CONTRIBUTIONS

Pereira JEG, Gomes JM, Santos EFS, Costa AS, Adami F, Figueiredo FWS, Sorpreso ICE and Abreu LC developed the study design and methodology. Pereira JEG, Gomes JM, Sorpreso ICE, Figueiredo FWS, Adami F and Abreu LC were involved with the data management. Pereira JEG, Santos EFS, Costa AS, Figueiredo FWS and Sorpreso ICE were involved with the data analyses. Pereira JEG, Santos EFS, Costa AS, Sorpreso ICE and Abreu LC drafted the manuscript. Pereira JEG, Gomes JM, Figueiredo FWS and Sorpreso ICE were involved in editing the manuscript. All authors read and approved the final version of the manuscript.

## Figures and Tables

**Table 1:** Socioeconomic characteristics of the physicians and other health professionals interviewed in the Health Care System for Women and Children (SASMC) in the state of Acre, Brazil, in 2017.

Characteristics	Physicians		Other health professionals		*p*-value*
	(n=76; 39.8%)		(n=120; 61.2%)		
	n	% (95% CI)	n	% (95% CI)	
Sex					
Male	28	20.2 (12.9; 27.5)	24	36.8 (25.9; 47.8)	0.01
Female	48	79.8 (72.5; 87.1)	95	63.2 (52.1; 74.1)	
Stable union					
No	23	47.4 (38.1; 56.6)	54	31.9 (21.0; 42.9)	0.04
Yes	49	52.6 (43.3; 61.9)	60	68.1 (57.1; 79.0)	
Children					
No	18	38.1 (29.3; 47.0)	45	24.0 (14.2; 33.8)	0.04
Yes	57	61.9 (53.0; 70.7)	73	76.0 (66.2; 85.8)	
Income					
Up to 10 times the normal wage	11	20.9 (13.2; 28.6)	87	84.7 (76.3; 93.1)	<0.001
Above 10 times the normal wage	61	79.1 (71.4; 86.8)	23	15.3 (6.9; 23.7)	
	Median (95% CI)		Median (95% CI)		*p*-value**
Age	40 (38; 43)		36 (34; 39)		0.006
Number of children	2 (1;2)		2 (2;2)		0.15

* Chi-square;

** Mann-Whitney (as assessed by interval estimation and 95% confidence intervals of proportions).

**Table 2:** Knowledge of HPV among physicians and other health professionals interviewed in the Health Care System for Women and Children (SASMC) in the state of Acre, Brazil, in 2017.

Knowledge of HPV and HPV vaccine	Total		Physicians	Other health professionals	RP	*p*-value*
	% (95% CI)	N	%	N		
Do you know what HPV is?	96.9 (93.1; 98.7)	190	98.7 (75)	95.8 (115)	0.93 (0.85; 1.01)	0.11
Is HPV a virus?	98.0 (94.5; 99.3)	192	98.7 (75)	97.5 (117)	0.95 (0.90; 1.00)	0.08
Is HPV a sexually transmitted disease?	98.0 (94.5; 99.3)	192	98.7 (75)	97.5 (117)	0.97 (0.93; 1.01)	0.15
Can HPV cause cervical cancer?	98.0 (94.5; 99.3)	192	100.0 (76)	96.7 (116)	0.96 (0.92; 1.01)	0.14
Can HPV cause changes in the Pap smear test?	93.4 (88.7; 96.3)	183	97.4 (74)	90.8 (109)	0.94 (0.84; 1.04)	0.24
Is cervical cancer a major cancer in women?	91.3 (86.3; 94.7)	179	97.4 (74)	87.5 (105)	0.88 (0.80; 0.97)	0.01
Can smoking increase the risk of cervical cancer?	71.9 (65.0; 78.0)	141	79.0 (60)	67.5 (81)	0.98 (0.79; 1.20)	0.83
Does the HPV vaccine prevent cervical cancer?	87.2 (81.6; 91.4)	171	90.8 (69)	85.0 (102)	0.91 (0.78; 1.05)	0.21
Should the HPV vaccine be given before the first sexual intercourse?	93.9 (89.3; 96.7)	184	93.4 (71)	94.2 (113)	1.00 (0.93; 1.2)	0.46
Can the HPV vaccine be given to people who have had sex?	74.0 (67.1; 79.9)	145	75.0 (57)	73.3 (88)	1.01 (0.82; 1.26)	0.86
Can the HPV vaccine be harmful to your health?	77.6 (70.9; 83.1)	152	75.0 (57)	79.2 (95)	1.15 (0.93; 1.41)	0.19
Can the HPV vaccine cause HPV infection?	75.0 (68.2; 80.8)	147	84.2 (64)	69.2 (83)	0.93 (0.76; 1.12)	0.43
Is the HPV vaccine provided by the government?	94.4 (89.9; 97.0)	185	92.1 (70)	95.8 (115)	0.98 (0.91; 1.05)	0.61
Is the HPV vaccine part of the girls' immunization records?	73.0 (66.1; 78.9)	143	71.1 (54)	74.2 (89)	0.91 (0.74; 1.12)	0.40
Are 3 doses required for complete vaccination?	61.2 (54.0; 68.0)	120	61.8 (47)	60.8 (73)	0.82 (0.61; 1.11)	0.20
Does the HPV vaccine decrease the chance of having genital warts?	71.9 (65.0; 78.0)	141	81.6 (62)	65.8 (79)	0.85 (0.65; 1.10)	0.22
Does the HPV vaccine decrease the chance of having changes in the Pap smear test?	80.6 (74.2; 85.8)	158	85.5 (65)	77.5 (93)	0.87 (0.71; 1.07)	0.21

RP: Ratio of proportions;

* Poisson regression adjusted for sex, stable union, age, and income was significant at *p*<0.05.

**Table 3:** Barriers, acceptability, and personal antecedents related to the HPV vaccine among physicians and other health professionals interviewed in the Health Care System for Women and Children (SASMC) in the state of Acre, Brazil, in 2017.

Domains	Total		Physicians	Other health professionals	RP	*p*-value*
	% (95% CI)	N	%	N		
Barriers						
Do you think the HPV vaccine will stimulate the onset of sexual activity at an earlier age?	95.4 (91.2; 97.7)	187	96.1 (73)	95.0 (114)	0.99 (0.93; 1.06)	0.98
Do you think that you still need to use a condom after HPV vaccination?	98.5 (95.2; 99.6)	193	100 (76)	97.5 (117)	0.94 (0.88; 1.01)	0.14
Do you think that you still need to have a Pap smear test after HPV vaccination?	99.0 (96.0; 99.8)	194	100 (76)	98.3 (118)	0.95 (0.88; 1.02)	0.16
Acceptability						
Do you know anyone who has already received the HPV vaccine?	74.0 (67.1; 79.9)	145	75.0 (57)	73.3 (88)	0.89 (0.71; 1.10)	0.29
Have you received the HPV vaccine yet?	10.2 (6.5; 15.5)	20	10.5 (8)	10.0 (12)	0.80 (0.28; 2.27)	0.68
Would you recommend the HPV vaccine for a child, friend, or relative?	93.4 (88.7; 96.3)	183	93.4 (71)	93.3 (112)	1.04 (0.96; 1.13)	0.34
Female personal history						p**
Have you ever had an abnormal Pap smear?	20.3 (14.2; 28.0)	29	25.0 (12)	17.9 (17)	0.71 (0.29; 1.74)	0.46
Have you ever had cervical cancer?	2.1 (0.5; 6.5)	3	2.08 (1)	2.11 (2)	1.55 (0.24; 10.02)	0.64
Have you ever had genital warts?	5.6 (2.6; 11.1)	8	12.5 (6)	2.1 (2)	0.17 (0.03; 1.12)	0.07

RP: Ratio of proportions;

* Poisson regression adjusted by sex, stable union, and age

** Poisson regression adjusted for stable union and age

**Table 4:** Clinical knowledge of the HPV vaccine among physicians and other health professionals interviewed in the Health Care System for Women and Children (SASMC) in the state of Acre, Brazil, in 2017.

Domains	Total		Physicians	Other health professionals	RP	*p*-value*
	% (95% CI)	N	%	N		
Can patients living with HIV take the vaccine?	48.5 (41.3; 55.7)	95	56.6 (43)	43.3 (52)	0.76 (0.52; 1.11)	0.17
Do I feel confident indicating HPV vaccination for patients?	79.1 (72.6; 84.4)	155	84.2 (64)	75.8 (91)	0.87 (0.73; 1.05)	0.15
Do I feel confident giving information about HPV to patients?	67.2 (60.0; 73.6)	131	73.7 (56)	62.5 (75)	0.78 (0.60; 1.02)	0.08
Can pregnant women take the vaccine?	39.8 (33.0; 47.0)	78	39.5 (30)	40.0 (48)	0.88 (0.54; 1.44)	0.63

RP: Ratio of proportions;

* Poisson regression adjusted for sex, stable union, age, and income
